# Application of artificial intelligence in the diagnosis of scaphoid fractures: impact of automated detection of scaphoid fractures in a real-life study

**DOI:** 10.1007/s11547-025-02028-5

**Published:** 2025-08-23

**Authors:** Ana Isabel Hernáiz Ferrer, Chandra Bortolotto, Luisa Carone, Emma Maria Preda, Cristina Fichera, Alice Lionetti, Giulia Gambini, Eleonora Fresi, Federico Alberto Grassi, Lorenzo Preda

**Affiliations:** 1 Diagnostic Imaging and Radiotherapy Unit, Department of Clinical, Surgical, Diagnostic, and Pediatric Sciences, University of Pavia, 27100 Pavia, Italy; 2 Radiology Institute, Fondazione IRCCS Policlinico San Matteo, 27100 Pavia, Italy; 3 SSD Biostatistica E Clinical Trial Center, Direzione Scientifica, Fondazione IRCCS Policlinico San Matteo, 27100 Pavia, Italy; 4 Department of Clinical, Orthopedics and Traumatology Clinic, Surgical, Diagnostic and Pediatric Sciences, IRCCS Policlinico San Matteo Foundation, 27100 Pavia, Italy

**Keywords:** Scaphoid bone, Wrist fractures, Radiographs, Artificial intelligence

## Abstract

**Purpose:**

We evaluated the diagnostic performance of two AI software programs (BoneView and RBfracture) in assisting non-specialist radiologists (NSRs) in detecting scaphoid fractures using conventional wrist radiographs (X-rays).

**Methods:**

We retrospectively analyzed 724 radiographs from 264 patients with wrist trauma. Patients were classified into two groups: Group 1 included cases with a definitive diagnosis by a specialist radiologist (SR) based on X-rays (either scaphoid fracture or not), while Group 2 comprised indeterminate cases for the SRs requiring a CT scan for a final diagnosis. Indeterminate cases were defined as negative or doubtful X-rays in patients with persistent clinical symptoms. The X-rays were evaluated by AI and two NSRs, independently and in combination. We compared their diagnostic performances using sensitivity, specificity, area under the curve (AUC), and Cohen’s kappa for diagnostic agreement.

**Results:**

Group 1 included 174 patients, with 80 cases (45.97%) of scaphoid fractures. Group 2 had 90 patients, of which 44 with uncertain diagnoses and 46 negative cases with persistent symptoms. Scaphoid fractures were identified in 51 patients (56.67%) in Group 2 after further CT imaging. In Group 1, AI performed similarly to NSRs (AUC: BoneView 0.83, RBfracture 0.84, NSR1 0.88, NSR2 0.90), without significant contribution of AI to the performance of NSRs. In Group 2, performances were lower (AUC: BoneView 0.62, RBfracture 0.65, NSR1 0.46, NSR2 0.63), but AI assistance significantly improved NSR performance (NSR2 + BoneView AUC = 0.75, *p* = 0.003; NSR2 + RBfracture AUC = 0.72, *p* = 0.030). Diagnostic agreement between NSR1 with AI support and SR was moderate (kappa = 0.576), and substantial for NSR2 (kappa = 0.712).

**Conclusions:**

AI tools may effectively assist NSRs, especially in complex scaphoid fracture cases.

## Introduction

The scaphoid bone plays a crucial role in maintaining wrist stability and enabling proper wrist motion, making it among the most important of the carpal bones. Scaphoid fractures are the most common type of carpal fracture, accounting for approximately 70% of these injuries [1]. Diagnosing these fractures is challenging for both clinicians and radiologists, as it is estimated that up to 20% of scaphoid fractures may not be visible on initial plain radiographs [2]. This percentage may be even higher among non-specialized radiologists (NSRs) who lack experience in musculoskeletal imaging [3]. Misdiagnosing occult scaphoid fractures can result in severe complications, including non-union, osteonecrosis, degenerative carpal osteoarthritis, and scaphoid non-union advanced collapse (SNAC) [4]. In indeterminate cases, where clinical suspicion of a fracture persists despite a negative radiology report or in the case of a doubtful report, it is crucial to obtain follow-up radiographs after 7–14 days and to consider second-line imaging, such as magnetic resonance imaging (MRI) or computed tomography (CT). This approach helps to prevent misdiagnosis but is also costly, time-consuming, and may not always be feasible depending on the local healthcare context [5].

Recent advancements in artificial intelligence (AI) have led to the development of software designed to assist radiologists in diagnosing bone fractures on plain radiographs [6]. Examples of these AI tools include BoneView, developed by Gleamer, and RBfracture, developed by Radiobotics. These technologies have already been integrated into routine clinical practice, enhancing the detection rate of fractures [7]. However, the full extent of their utility, particularly in the complex scenario of scaphoid fractures, is still under evaluation.

In this study, we evaluated the contribution of the AI tools BoneView and RBfracture, to the diagnostic performance of NSRs in detecting scaphoid fractures on plain radiographs in patients with wrist trauma. Additionally, we compared the performance characteristics of these two AI software in this task.

## Methods

This study was approved by our Institutional Review Board and carried out in accordance with the ethical standards of the Helsinki Declaration. All patients provided written informed consent for image acquisition and anonymized use of their data.

### Study population

We enrolled 264 consecutive patients (764 radiographs) who presented with hand or wrist trauma to the Emergency Department of our institution between January 2014 and May 2023.

We included patients over 18 years old with clinical suspicion of scaphoid fracture, determined through clinical evaluation (eg., pain, swelling and tenderness localized in the anatomical snuffbox), who underwent plain radiographs as the initial imaging investigation. In cases of uncertain diagnosis after the initial imaging, only patients who underwent second imaging to reach a definitive diagnosis were included.

Exclusion criteria included patients with advanced arthrosis and complete fusion of the carpal bones, pediatric patients whose carpal bones are not completely ossified depending on bone age, and cases with a history of previous carpal surgery or chronic/consolidated scaphoid fractures. These cases were excluded following a review by a specialist radiologist with over 10 years of expertise in musculoskeletal and emergency imaging.

### AI software used in the study

We used two artificial intelligence packages: BoneView (Gleamer, Paris, France) and RBfracture (Radiobotics, Copenhagen, Denmark). These deep learning AI systems, based on a deep convolutional neural network (DCNN) using the “Detectron2” framework, assist healthcare professionals in detecting potential bone fractures on radiographs and reducing missed fractures. They process radiographs in DICOM format as input and produce an output image that highlights the detected fractures with a bounding box. Both programs classify radiographs as “Positive,” “Doubtful,” or “Negative” for fractures, using a solid bounding box where a fracture is detected and a dashed bounding box where a fracture is possible (“Doubtful”). These AI algorithms are currently licensed as support tools for fracture detection both in North America and Europe. We will refer to Boneview as Software 1 (SW1) and RBfracture as Software 2 (SW2) throughout the text. Additional information on Boneview and RBfracture is provided in Supplementary Materials.

### Study design and procedures

**Fig. 1:**
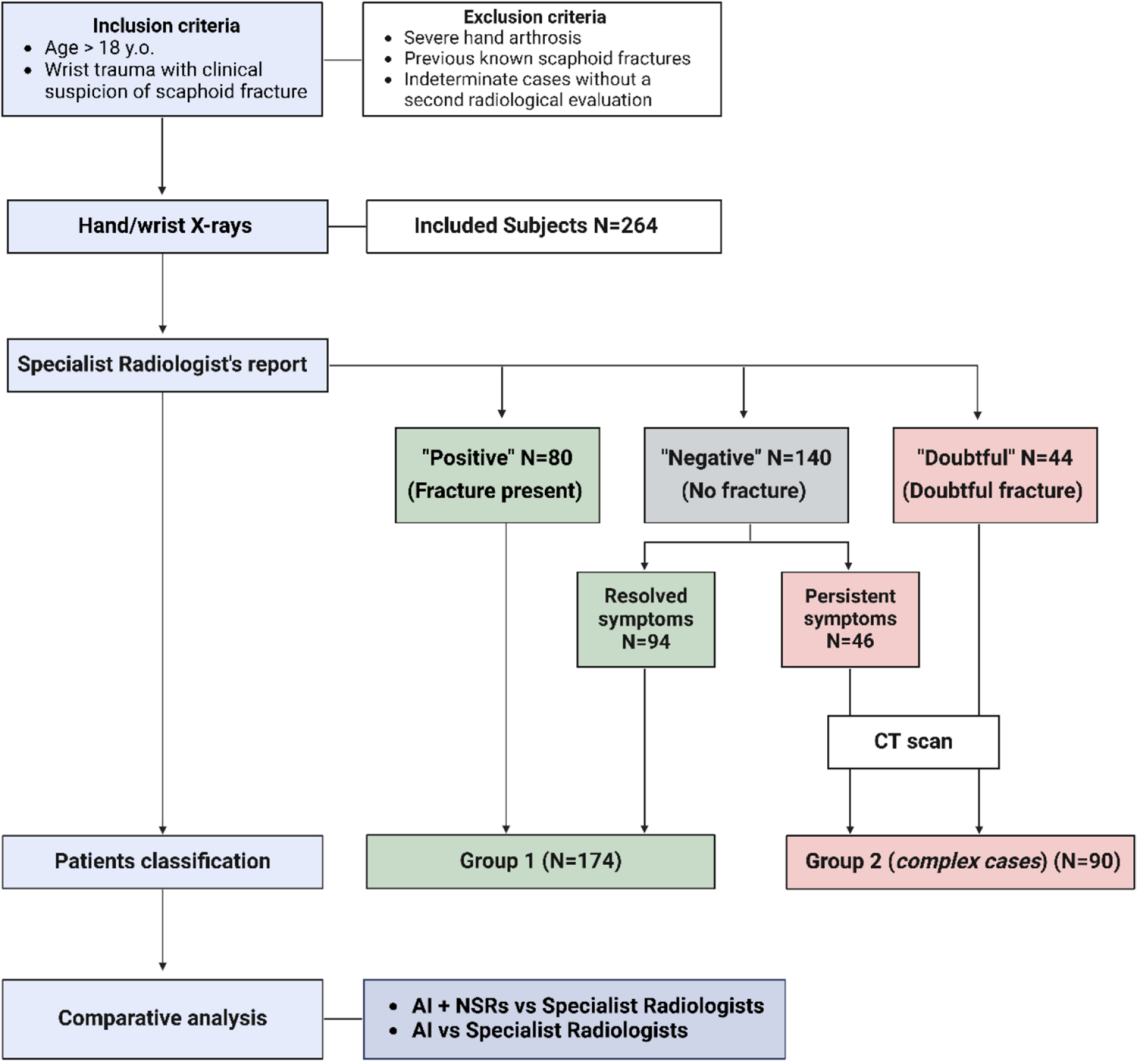
Flowchart of the study design. Abbreviations: y.o., years old; NSR, non-specialized radiologists. Figure created with Biorender

This study was designed as a single-site, longitudinal, retrospective study. Data collection and analysis took place between November 2023 and June 2024. Gleamer and Radiobotics provided free trial access to their AI software for this study. The flowchart in Fig. 1 outlines the study design and analysis.

Radiographs of enrolled patients were classified as ‘Positive,’ ‘Negative,’ or ‘Doubtful’ for scaphoid fractures based on original reports extracted from the radiology information system (RIS). These reports were validated at the time of acquisition by specialist radiologists (SRs) with at least five years of experience in musculoskeletal and emergency imaging. Patients were divided into two groups. Group 1 included cases with a definite radiological diagnosis (either the presence or absence of a scaphoid fracture) based on reports labeled ‘Positive’ or ‘Negative.’ In this group, the initial X-ray assessment served as the reference standard for the statistical analyses. Group 2, the *complex cases*, included those that required a CT scan at the time of the original evaluation to reach a final diagnosis. This group comprised cases labeled as ‘Doubtful’ and a portion of the ‘Negative’ cases with persistent clinical suspicion of fracture, where second imaging was performed due to a clinical-radiological discrepancy. In this group, the CT evaluation served as the reference standard for the statistical analyses.

**Fig. 2:**
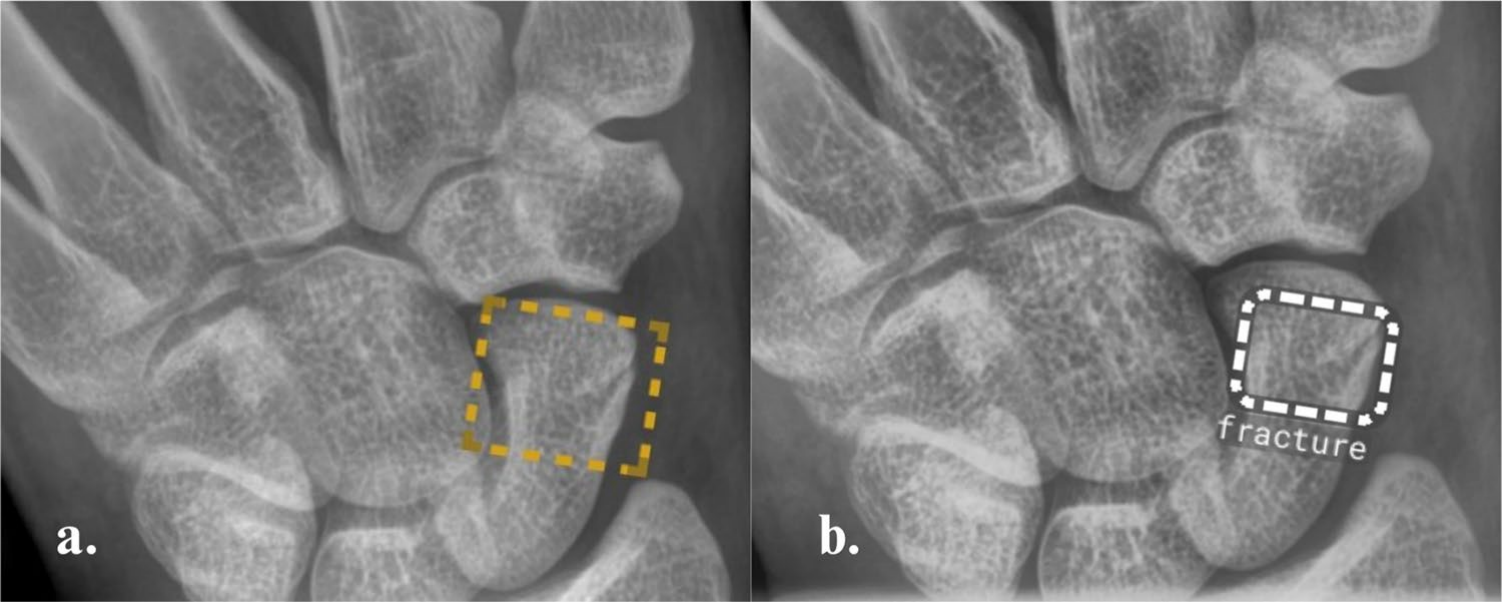
Posteroanterior radiograph of the wrist of a male patient. A scaphoid fracture is marked with a dashed bounding box, indicating a possible fracture (‘Doubt’), by the AI software SW1 (**a**) and SW2 (**b**)

**Fig. 3:**
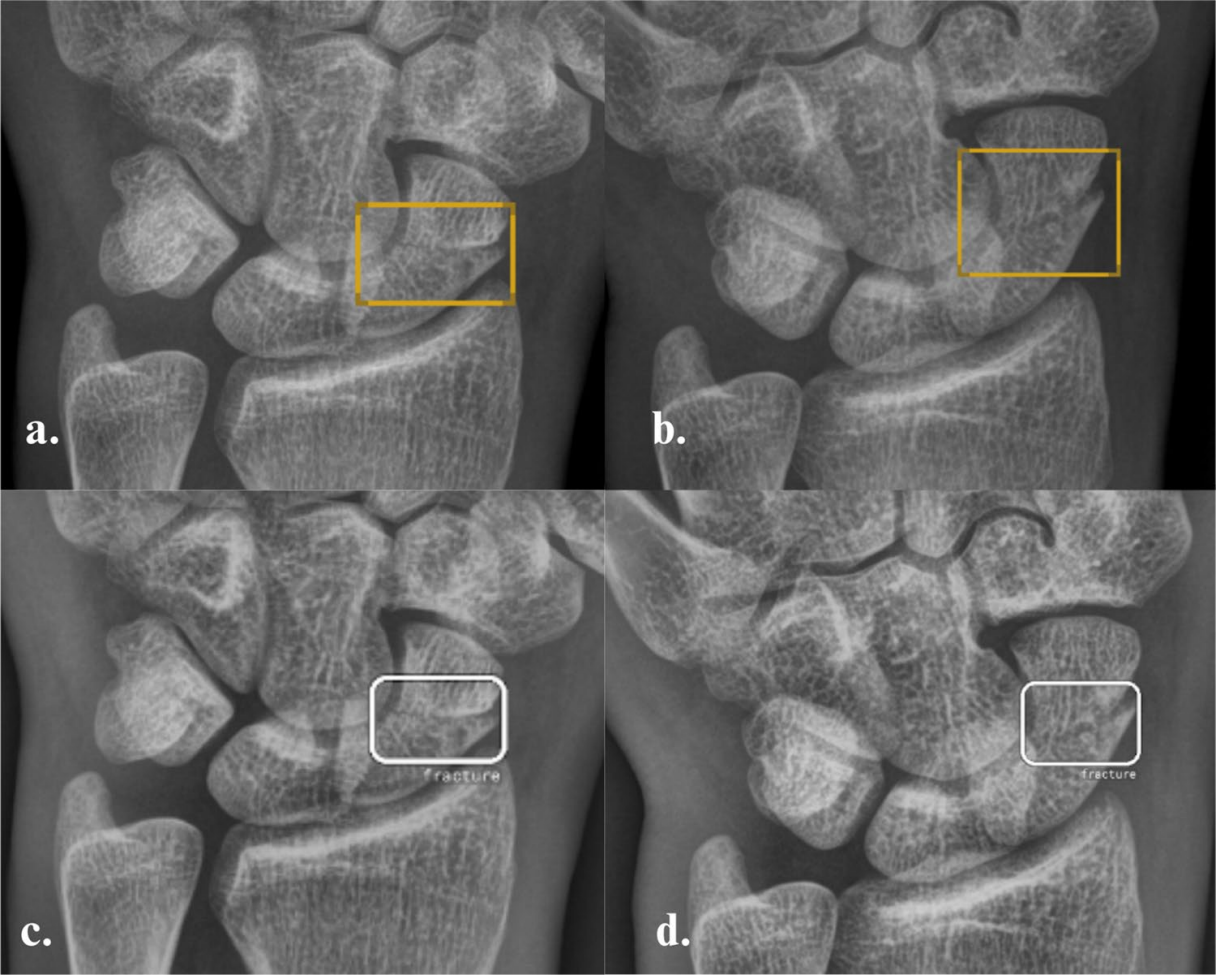
Posteroanterior and ulnar deviation radiographs of the wrist of a male patient from Group 1. The scaphoid fracture is correctly marked as ‘Positive’ with a bounding box by the AI software SW1 (**a**, **b**) and SW2 (**c**, **d**)

**Fig. 4:**
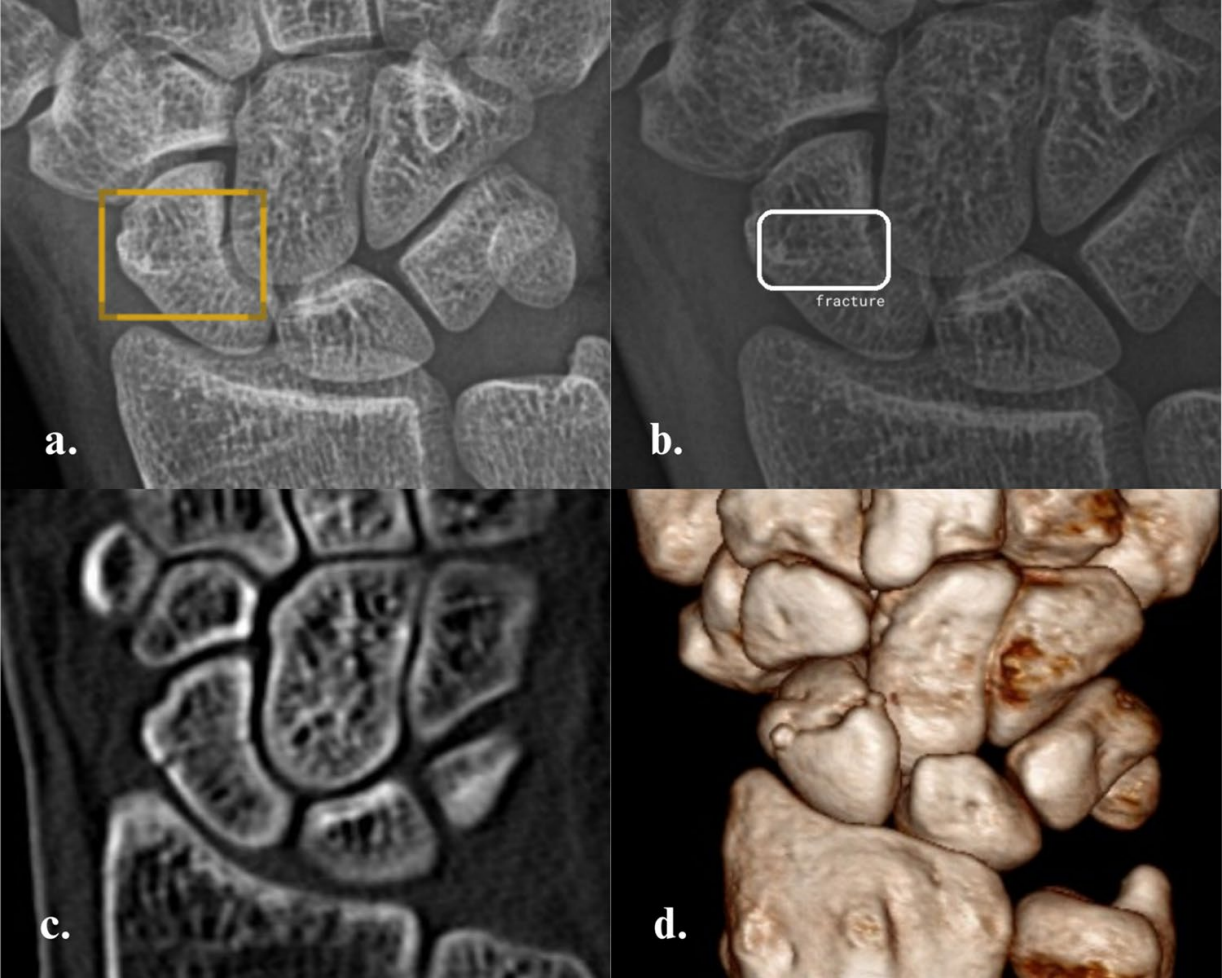
Occult scaphoid fracture of a male patient from Group 2. The fracture is correctly marked as 'Positive' by both AI software programs on a posteroanterior ulnar deviation radiograph of the wrist [SW1 (**a**) and SW2 (**b**)]. The fracture was then confirmed with a CT scan (**c**) and Coronal Volume Rendering (**d**)

Radiographs from both groups were analyzed by the AI software and by two NSRs (with < 5 months of experience in musculoskeletal and emergency radiology). The NSRs initially produced results without AI assistance, followed by a second set using AI results. To avoid bias, a washout period of at least two weeks was applied between analyses, and radiographs were randomized for each session. Both AI and NSRs classified cases as ‘Positive,’ ‘Negative,’ or ‘Doubtful.’ Figs. 2, 3 and 4 show examples of radiological images analyses.

Ulnar-deviation radiographs (UDR) were available for a subset of 145 patients, performed at the radiologist’s request for radioprotection.

### Statistical analysis

The primary objective of the study was to evaluate the *diagnostic performance* of two NSRs, with and without support from two AI tools (SW1 and SW2), in detecting scaphoid fractures on plain radiographs. We also compared the accuracy of the two AI software tools in this task. Additionally, we assessed the *diagnostic agreement* among our gold standard and NSRs (with and without AI support) and between the two AI software. Finally, we evaluated the *impact of the specific UDR* on the diagnostic performance of both AI algorithms.

The comparative gold standards (ground truth) used for assessing diagnostic performance were the reports stored in our RIS and validated by specialist radiologists (SRs); for Group 1 consisted of radiograph reports, while for Group 2 consisted of CT scan reports.

Diagnostic performance was assessed by measuring sensitivity (Se), specificity (Sp), area under the curve (AUC), odds ratio (OR), positive predictive value (PPV), and negative predictive value (NPV). The corresponding 95% confidence interval (95% CI) was determined for each diagnostic performance measurement. The McNemar test was used to compare sensitivity and specificity, with *p*-values less than 0.05 considered statistically significant. AUC refers to the area under the receiver operating characteristic (ROC) curve, which plots the true positive rate against the false positive rate, measuring the overall ability of a model to distinguish between classes, with values ranging from 0.5 (no discrimination) to 1 (perfect discrimination). Diagnostic agreement was evaluated using Cohen’s kappa coefficient. For the interpretation of Cohen’s kappa values, we follow these thresholds: < 0.00, poor agreement; 0.00–0.20, slight agreement; 0.21–0.40, fair agreement; 0.41–0.60, moderate agreement; 0.61–0.80, substantial agreement; 0.81–1.00, almost perfect agreement.

Data analysis was performed using Stata software (Release 18 or later, StataCorp, College Station, TX, USA).

## Results

### Demographics and groups characteristics

The study included 264 patients (164 males, 62.12%) aged 18 to 100 years (mean age 49.28 ± 20.69 years), with a total of 764 radiographs.

Based on SRs' reports, we identified 80 “Positive” cases of scaphoid fractures (30.30%), 140 “Negative” cases (53.03%), and 44 “Doubtful” cases (16.67%). Group 1 comprised 174 cases, with 80 fractures detected (45.98%). All “Doubtful” cases, along with 46 of the “Negative” cases with persistent clinical symptoms, underwent CT scans for diagnostic confirmation and were classified in Group 2 (*n* = 90). After the second imaging evaluation, a scaphoid fracture was identified in 51 patients (56.67%) in Group 2, including 29 (32.22%) patients whose original radiographs were initially reported as negative (occult fractures) and 22 patients (24.44%) whose original radiographs were initially reported as “doubtful”. Figure 1 illustrates the subject classification.

A total of 131 fractures were identified and classified according to the “AO/OTA Scaphoid 72” (Orthopedic Trauma Association) classification into subtypes: 15 proximal fractures (11.45%), 77 medial fractures (58.77%), and 39 distal fractures (29.77%). Of these, 128 were non-comminuted (97.7%) and 3 were comminuted (2.3%).

### Diagnostic performance of AI software and non-specialized radiologists in group 1

The diagnostic performance results of the AI tools and NSRs for Group 1 are illustrated in Table 1.

**Table 1:** Performance of AI software and non-specialized radiologists (NSR1 and NSR2) in Group 1

Group 1	Sensitivity (95% CI)	Specificity (95% CI)	PPV (95% CI)	NPV (95% CI)	AUC (95% CI)	Odds Ratio (95% CI)
SW1	81.2% (71.0–89.1)*	85.1% (76.3–91.6)*	82.3% (72.1–90.0)	84.2% (75.3–90.9)	0.83 (0.78–0.89)	24.76 (11.20–54.74)
SW2	70.0% (58.7–79.7)*	97.9% (92.5–99.7)*	96.6% (88.1–99.6)	79.3% (70.8–86.3)	0.84 (0.79–0.89)	0.31 (0.22–0.43)
NSR1	81.2% (71.0–89.1)	95.7% (89.5–98.8)	94.2% (85.8–98.4)	85.7% (77.5–91.8)	0.88 (0.84–0.93)	97.50 (31.86–294.65)
NSR1 with SW1	87.5% (78.2–93.8)	87.2% (78.8–93.2)*	85.4% (75.8–92.2)	89.1% (80.9–94.7)	0.87 (0.82–0.92)	47.83 (19.64–116.48)
NSR1 with SW2	78.8% (68.2–87.1)	96.8% (91.0–99.3)	95.5% (87.3–99.1)	84.3% (76.0–90.6)	0.88 (0.83–0.93)	112.42 (33.16–375.18)
NSR2	82.5% (72.4–90.1)	97.9% (92.5–99.7)	97.1% (89.8–99.6)	86.8% (78.8–92.6)	0.90 (0.86–0.95)	216.86 (51.84-NP)
NSR2 with SW1	88.8% (79.7–94.7)	91.5% (83.9–96.3)*	89.9% (81.0–95.5)	90.5% (82.8–95.6)	0.90 (0.86–0.95)	84.81 (31.43–228.77)
NSR2 with SW2	88.6% (79.5–94.7)	94.7% (88.0–98.3)	93.3 (85.1–97.8)	90.8 (83.3–95.7)	0.92 (0.87–0.96)	138.44 (45.22–421.43)

The statistically significant differences (*P* value < 0.05) in diagnostic performance between the two AI programs, and between NSRs with and without AI assistance, are highlighted in bold and marked with an asterisk

SW1, Software 1 (Boneview); SW2, Sofware 2 (RBfracture); *PPV* positive predictive value; *NPV* negative predictive value; *AUC* area under the curve; *OR* odds ratio; *CI* confidence interval; *NP* not provided

Sensitivity, specificity, and AUC were 81.2%, 85.1%, and 0.83 for SW1; 70%, 97.9%, and 0.84 for SW2; 81.2%, 95.7%, and 0.88 for NSR1; and 82.5%, 97.9%, and 0.90 for NSR2, respectively. SW1 showed statistically higher sensitivity compared to the SW2 (*p* = 0.035). Conversely, the specificity of SW2 was significantly higher than that of SW1 (*p* = 0.002). The AUC for both software and NSRs did not differ significantly (NSR1 vs SW1: *p* = 0.67; NSR1 vs SW2: *p* = 0.77; NSR2 vs SW1: *p* = 0.97; NSR2 vs SW2: *p* = 0.45; SW1 vs SW2: *p* = 0.80). Both software programs slightly improved the diagnostic sensitivity of the NSRs, though the difference was not statistically significant (NSR1 + SW1 vs. NSR1: *p* = 0.18; NSR1 + SW2 vs. NSR1: *p* = 0.75; NSR2 + SW1 vs. NSR2: *p* = 0.13; NSR2 + SW2 vs. NSR2: *p* = 0.18). However, SW1 modestly reduced the specificity of both radiologists (NSR1: *p* = 0.057; NSR2: *p* = 0.031). The AUCs of the NSRs with the assistance of AI did not change in a statistically significant fashion (NSR1 + SW1 vs. NSR1: *p* = 0.67; NSR1 + SW2 vs. NSR1: *p* = 0.77; NSR2 + SW1 vs. NSR2: *p* = 0.97; NSR2 + SW2 vs. NSR2: *p* = 0.45).

### Diagnostic performance of AI software and non-specialized radiologists in group 2

The diagnostic performance results of the AI tools and NSRs for Group 2 are shown in Table 2.

**Table 2:** Performance of AI software and non-specialized radiologists (NSR1 and NSR2) in Group 2

Group 2	Sensitivity (95% CI)	Specificity (95% CI)	PPV (95% CI)	NPV (95% CI)	AUC (95% CI)	Odds Ratio (95% CI)
SW1	60.8% (46.1–74.2)	64.1% (47.2–78.8)	68.9% (53.4–81.8)	55.6% (40.0–70.4)	0.62 (0.52–0.73)	2.77 (1.18–6.51)
SW2	47.1% (32.9–61.5)	82.1% (66.5–92.5)	77.4% (58.9–90.4)	54.2% (40.8–67.3)	0.65 (0.55–0.74)	4.06 (1.54–10.65)
NSR1	51.0% (36.6–65.2)	41.0% (25.6–57.9)	53.1% (38.3–67.5)	39.0% (24.2–55.5)	0.46 (0.36–0.56)	0.72 (0.31–1.67)
NSR1 with SW1	72.5% (58.3–84.1)*	48.7% (32.4–65.2)	64.9% (51.1–77.1)	57.6% (39.2–74.5)	0.61 (0.51–0.71)*	2.51 (1.05–6.00)
NSR1 with SW2	66.7% (52.1–79.2)	53.8% (37.2–69.9)	65.4% (50.9–78.0)	55.3% (38.3–71.4)	0.60 (0.50–0.71)*	2.33 (1.00–5.46)
NSR2	68.6% (54.1–80.9)	56.4% (39.6–72.2)	67.3% (52.9–79.7)	57.9% (40.8–73.7)	0.63 (0.52–0.73)	2.89 (1.20–6.69)
NSR2 with SW1	84.3% (71.4- 93.0)*	66.7% (49.8–80.9)	76.8% (63.6–87.0)	76.5% (58.8–89.3)	0.75 (0.66–0.85)*	10.75 (3.98–29.01)
NSR2 with SW2	80.4% (66.9–90.2)*	64.1% (47.2–78.8)	74.5% (61.0–85.3)	71.4% (53.7–85.4)	0.72 (0.63–0.82)*	7.32 (2.85–18.77)

The statistically significant differences (*P* value < 0.05) in diagnostic performance between the two AI programs, and between NSRs with and without AI assistance, are highlighted in bold and marked with an asterisk

SW1, Software 1 (Boneview); SW2, Sofware 2 (RBfracture); *PPV* positive predictive value; *NPV* negative predictive value; *AUC* area under the curve; *OR* odds ratio; *CI* confidence interval

SW1 achieved a sensitivity of 60.8%, while SW2 reached 47.1%; this difference was not statistically significant (*p* = 0.167). SW2 maintained a specificity consistent with its performance in Group 1 (82.1%), and higher than that of SW1 (64.1%), with a trend toward statistical significance (*p* = 0.065). The AUC for both software tools were similar (SW1 AUC = 0.62; SW2 AUC = 0.65; *p* = 0.72).

**Fig. 5:**
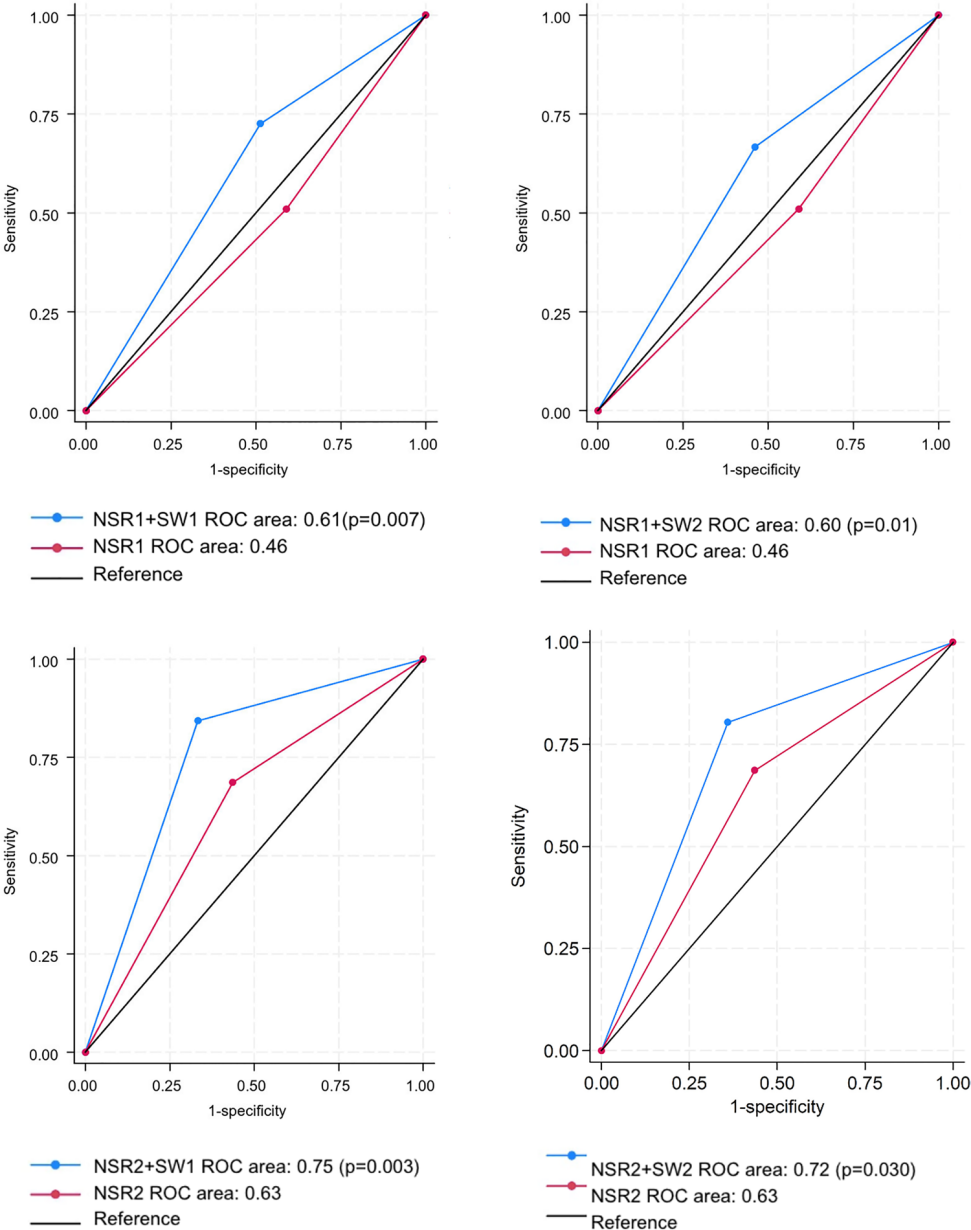
Diagnostic accuracy of non-specialist radiologist (NSR) with AI in Group 2. The charts in the top row represent the diagnostic accuracy of NSR1 with the AI software BoneView (SW1, on the left) and RBfracture (SW2, on the right). The charts in the bottom row represent the same for NSR2

In this group, sensitivity, specificity and diagnostic accuracy of both NSRs were markedly inferior to that in Group 1 (NSR1: Se = 51%, Sp = 41%, AUC = 0.46; NSR2: Se = 68.6%, Sp = 56.4%, AUC = 0.63). Both AI programs improved the overall diagnostic performance of the NSRs. Notably, sensitivity significantly increased with SW1 in both NSRs, by 21.5% in NSR1 (72.5%, *p* = 0.013) and by 15.7% in NSR2 (84.3%, *p* = 0.008) and with SW2 in NSR2 by 11.8% (80.4%, *p* = 0.031). While specificity increased for both NSRs, the changes were not statistically significant (NSR1 + SW1 vs. NSR1: *p* = 0.51; NSR1 + SW2 vs. NSR1: *p* = 0.18; NSR2 + SW1 vs. NSR2: *p* = 0.29; NSR2 + SW2 vs. NSR2: *p* = 0.51). Diagnostic accuracy with AI assistance increased in a statistically significant fashion for both NSR1 (with SW1 AUC = 0.61, *p* = 0.007; with SW2 AUC = 0.60, *p* = 0.01) and NSR2 (with SW1 AUC = 0.75, *p* = 0.003; with SW2 AUC = 0.72, *p* = 0.030). Diagnostic accuracy of NSRs with and without AI in Group 2 are represented in Fig. 5.

### Diagnostic agreement

The diagnostic agreement between NSRs assisted by AI and specialist radiologists was moderate for NSR1 (NSR1 with SW1 kappa = 0.576; NSR1 with SW2 kappa = 0.583) and substantial for NSR2 (NSR2 with SW1 kappa = 0.712; NSR2 with SW2 = 0.711). The diagnostic agreement between the two software tools was moderate (kappa = 0.544). Results are shown in Table 3.

**Table 3:** Diagnostic agreement between non-specialized radiologists (NSRs) with and without AI support and specialist radiologists (SR), and between the two AI tools alone

	Cohen’s Kappa	Agreement (%)
SW1 and SW2	0.544	77.65
NSR1 and SR	0.492	74.62
NSR1 with SW1 and SR	0.576	78.79
NSR1 with SW2 and SR	0.583	79.17
NSR2 and SR	0.629	81.44
NSR2 with SW1 and SR	0.712	85.61
NSR2 with SW2 and SR	0.711	85.55

SW1, Software 1 (Boneview); SW2, Sofware 2 (RBfracture)

### Role of wrist radiography with ulnar-deviation view

Out of all 764 radiographs, 145 were specific projections with ulnar deviation. This projection was found to provide a high diagnostic specificity for both AIs (SW1 92%, SW2 89.3%), but did not improve the overall AI diagnostic performance.

## Discussion

In this study, we evaluated the impact of two commercially available and clinically validated AI software systems—Boneview by Gleamer and RBfracture by Radiobotics—on assisting non-experienced radiologists in detecting scaphoid fractures in a real-life setting. We also assessed the diagnostic performance of both AI tools independently, the diagnostic agreement between AI and human readers, and the role of dedicated projections, specifically the ulnar deviation radiograph. Additionally, we analyzed a subset of complex clinical cases where standard radiographs were insufficient for reliably detecting scaphoid fractures, necessitating the use of CT scans. Our results indicate that in definite cases of scaphoid fracture—where fractures were readily identifiable on standard X-rays by a specialist radiologist—both AI tools demonstrated good sensitivity, specificity, and AUC, comparable to non-specialized radiologists. However, in more complex cases, as expected, the diagnostic performance of both AI tools and non-specialist radiologists declined. Notably, AI support significantly increased the diagnostic confidence of non-specialized radiologists in these cases, suggesting potential implications for clinical practice.

There is limited data in the literature on the use of AI software for diagnosing scaphoid fractures. A recent systematic review and meta-analysis summarized findings from 10 clinical studies evaluating AI in scaphoid fracture detection, highlighting that AI generally achieves high sensitivity, specificity, and AUC, consistent with our results [3]. However, only one of these studies assessed clinically implemented AI software [8], while the others used convolutional neural networks (CNNs) specifically designed for scaphoid fractures, which may limit the generalizability and real-world applicability of their findings.

Few studies have directly compared AI diagnostic performance to that of human radiologists for scaphoid fracture detection, yielding mixed results. Langerhuizen et al. [9] and Hendrix et al. [10] found no significant difference between CNN-based AI models and orthopedic surgeons or radiologists, respectively. Conversely, Ozkaya et al. [11] reported that CNN performance was inferior to that of an orthopedic specialist. Across these three studies, AUC values ranged from 0.77 to 0.87, suggesting overall promising diagnostic accuracy. Our findings indicate that AI performed at a radiologist-level in detecting straightforward scaphoid fractures. However, in complex cases, the diagnostic performance of both AI and, in particular, non-specialized radiologists was significantly lower. Furthermore, the use of ulnar deviation radiographs did not impact AI diagnostic accuracy.

The impact of AI on clinicians’ diagnostic performance in the context of scaphoid fractures has been scarcely investigated. Hendrix et al. [12] found that AI assistance did not improve radiologists’ diagnostic accuracy but reduced their reading time. In contrast, Lee et al. [13] and Cohen et al. [8] demonstrated that AI support significantly enhanced non-specialized radiologists’ accuracy, reflected in improved AUC (from 0.75 to 0.85 for radiologist 1 and from 0.71 to 0.80 for radiologist 2) and sensitivity (from 76 to 88%), respectively. In our study, AI software generally improved radiologists' diagnostic performance in both simple and complex cases of scaphoid fractures. However, a statistically significant benefit was observed only in the subset of complex fractures. Even with AI support, diagnostic accuracy in these cases remained suboptimal, with the highest AUC reaching only 0.75. The diagnostic agreement between NSR1 with AI support and specialist radiologists was moderate, while for NSR2, it was substantial, suggesting that AI assistance may help reduce the performance gap between non-specialist and specialist radiologists in scaphoid fracture detection. These findings highlight AI’s potential to enhance confidence and reduce inter-reader variability, particularly where specialist radiologist availability is limited.

To our knowledge, this is the first study to compare two widely implemented AI tools designed for general fracture detection in the context of scaphoid fractures, rather than AI models specifically trained for this purpose. Both AI tools demonstrated similar performance, with Boneview exhibiting higher sensitivity and RBfracture demonstrating greater specificity. A potential clinical implication of these findings could be the use of Boneview in settings where prioritizing sensitivity is crucial (e.g., emergency departments), minimizing false negatives. Conversely, RBfracture may be more suitable for specialized musculoskeletal radiology settings, where its higher specificity could aid in resolving diagnostic uncertainties among expert radiologists.

This study has some limitations, including its retrospective design and relatively small sample size. Additionally, not all scaphoid fractures were confirmed by second-level imaging, such as CT scans. However, the study also has key strengths that distinguish it from previous research on AI-assisted scaphoid fracture detection. First, we tested commercially available AI software ready to be implemented into clinical practice, enhancing the real-world applicability of their findings. Unlike many prior studies, we evaluated AI both independently and in combination with human readers, also encompassing a set of *complex cases*, which included occult fractures. Furthermore, we focused on non-specialized radiologists as human readers, reflecting their frequent role in emergency departments. Rather than comparing AI to orthopedic surgeons—who typically rely on radiologist reports—or to specialized radiologists, who may not be the primary target for AI support, we assessed its impact on those most likely to use it in clinical practice. Unlike many studies that compare AI to non-radiologists (e.g., clinicians, surgeons), potentially overstating its effectiveness, our approach may be able to provide a more realistic clinical perspective.

In conclusion, this is the first study to evaluate the use of two commercially available AI software tools for non-specialized radiologists in the setting of scaphoid fracture detection. Our results demonstrate a positive impact of AI support on non-specialized radiologists’ diagnostic performance, particularly in complex cases. However, the diagnostic accuracy in challenging cases remains suboptimal. Further research should explore the cost-effectiveness of AI implementation from a health economics perspective and employ prospective study designs to assess its full clinical utility.
